# Quality of Life, Sexual Functioning, and Physical Functioning Following Perineal Reconstruction with the Lotus Petal Flap

**DOI:** 10.1245/s10434-020-08771-5

**Published:** 2020-07-02

**Authors:** Joke Hellinga, Martin W. Stenekes, Paul M. N. Werker, Moniek Janse, Joke Fleer, Boudewijn van Etten

**Affiliations:** 1 Department of Plastic Surgery, University Medical Center Groningen, University of Groningen, Groningen, The Netherlands; 2 Department of Health Sciences, Section Health Psychology, University Medical Center Groningen, University of Groningen, Groningen, The Netherlands; 3 Department of Surgical Oncology, University Medical Center Groningen, University of Groningen, Groningen, The Netherlands

## Abstract

**Background:**

Lotus petal flaps (LPF) may be used for the reconstruction of extralevator abdominoperineal defects that cannot be closed primarily. Limited data are available on how perineal reconstruction with the LPF impacts on patients’ quality of life (QoL), sexual functioning, and physical functioning.

**Methods:**

A cross-sectional study was performed following perineal reconstruction with the LPF. The QoL of patients having undergone LPF reconstruction was compared with a control group in which perineal defects were closed without flaps. Sexual and physical functioning (presence of perineal herniation and range of motion [ROM] of the hip joints) could only be evaluated in the LPF group. Psychometrically sound questionnaires were used. Physical functioning was evaluated subjectively with binary questions and objectively by physical examination.

**Results:**

Of the 23 patients asked to participate, 15 (65%) completed the questionnaires and 11 (47%) underwent physical examination. In the control group, 16 patients were included. There were no significant differences in QoL between the LPF and control groups. Within the LPF group, 33% of patients were sexually active postoperatively compared with 87% preoperatively. No perineal herniation was found. The ROM of the hip joints was bilaterally smaller compared with the generally accepted values.

**Conclusions:**

Conclusions should be made with care given the small sample size. Despite a supposedly larger resection area in the LPF group, QoL was comparable in both groups. Nonetheless, reconstruction seemed to affect sexual function and physical function, not hampering overall satisfaction.

Resection of advanced anal and rectal tumors by means of extralevator abdominoperineal excision (ELAPE) may lead to extensive soft tissue defects in the perineal area.^1^ Wound healing may be impaired due to the application of neoadjuvant radiotherapy and chemotherapy, especially in patients with advanced cancer and malnutrition.^1^,^2^ When resulting defects are too large for tension-free primary closure, reconstruction with a soft tissue flap should be performed.^2^,^3^ Such a reconstruction has the potential for bringing well-vascularized, non-irradiated soft tissue into the defects and is able to fill the dead space and minimizes the risk of fluid collections and infections.^1^,^2^,^4^,^5^ Our first choice of flaps following ELAPE is the lotus petal flap (LPF)^6^, a regional flap based on perforators of the internal and external pudendal vessels behind.^2^–^7^ This flap has been widely used for vulvar reconstruction over the past decades, yet the application for perineal reconstruction is relatively new as its use was reported for the first time in 2007.^6^,^8^–^10^

From research on vulvar reconstruction, we know that reconstructive surgery in this area may have a large impact on quality of life (QoL) and sexual functioning.^11^ However, little or no data are available on how perineal reconstruction with the LPF impacts on patients’ lives (i.e. QoL, sexual functioning, and physical functioning). Perineal herniation has been reported as a common complication (incidence rates vary from 1 to 26%) following ELAPE.^12^ Reconstruction with a soft tissue flap supposedly lowers the risk of perineal herniation, but currently there are no data available to support this statement.^13^ There are also no data available on the ROM of the ipsilateral hip joint after LPF, whereas it is likely that this procedure influences the function of the hip joint as the LPF donor site is located on the junction of the buttock and the upper thigh and is sometimes quite tight after primary closure.

Therefore, the primary aim of this study was to assess the postoperative QoL of patients who had undergone LPF reconstruction following ELAPE and compare this with a control group in whom primary closure of the perineal defect following abdominoperineal excision (APE) had been possible. The secondary aim was to assess postoperative sexual and physical functioning in both groups. Two aspects of physical functioning had our main interest; first, the presence of perineal herniation, and second, the range of motion (ROM) of the hip joint on the side where the LPF was harvested. Our hypothesis is that QoL in the reconstructive group is higher compared with the control group. Furthermore, we believe that sexual function will be influenced by the reconstruction, but physical functioning will barely be affected.

## Methods

### Patients and Procedures

The LPF group consisted of patients who underwent perineal reconstruction with the LPF at the University Medical Center Groningen, The Netherlands, between October 2011 and December 2015. Patients who were deceased at the time of follow-up and patients who were not able to read or write Dutch were excluded. Eligible patients received a package containing an information letter, an informed consent form, the questionnaire, and a return envelope. Patients who did not reply within 2 weeks were called by the first author, asking them if they had received the package and asking them to contemplate to return the informed consent and, if applicable, the questionnaire. After QoL data collection was completed, participating patients were asked to visit the outpatient clinic to be physically examined. They were asked to sign informed consent during the visit to the clinic.

The historical control group consisted of colorectal cancer patients who had undergone APE and radiotherapy between December 2011 and March 2013 and did not need reconstruction because of the limited size of the resulting defect after tumor resection allowing for primary closure. These patients were selected from a larger study performed by Janse et al.^14^ investigating QoL in the first 18 months after colorectal cancer.

### Measures

Data regarding patient demographics and treatment and disease characteristics were collected from the medical files.

QoL was assessed in both the LPF and the control group using two psychometrically sound questionnaires. The global health status scale and the five functional scales (i.e. physical, role, emotional, cognitive, and social functioning) of the European Organization for Research and Treatment of Cancer Quality of Life Questionnaire C30 (EORTC QLQ-C30) version 3.0 were used to assess general QoL.^15^ After transformation, the scores ranged from 0 to 100. In addition, the European Organization for Research and Treatment of Cancer Quality of Life Questionnaire CR29 (EORTC QLQ-CR29), which was specifically developed to assess the QoL of colorectal cancer patients, was used.^16^,^17^ This questionnaire consists of three functional scales (i.e. body image, anxiety, and weight). After transformation the scores also ranged from 0 to 100.

Sexual functioning and physical functioning were only assessed in the LPF group. The Female Sexual Function Index (FSFI) and the Male Sexual Function Index (MSFI) were used to assess sexual functioning of the female and male patients, respectively.^18^–^20^ Only the total score was used (maximum of 36 points). A total score > 26 is considered as normal sexual functioning.^21^ In addition, binary questions were used to assess whether or not patients (1) currently had a partner; (2) were sexually active before surgery; (3) were sexually active after surgery; and (4) had intercourse after surgery. Sexual activity was permitted 6 weeks postoperatively. Postoperative sexual activity was also assessed in the control group.

LPF-specific physical functioning was measured subjectively and objectively. Subjective physical functioning was measured by asking whether or not patients experienced (1) any perineal bulging, reflecting perineal herniation (during sitting, standing or walking); (2) limitations in the use of the left hip (during sitting or walking); (3) limitations in the use of the right hip (during sitting or walking); (4) other limitations during sitting; and (5) limitations during cycling. Objective physical functioning was measured by determination of the active ROM (flexion) in both hips (using a goniometer) according to the standard way of measuring of the American Academy of Orthopaedic Surgeons.^22^ Of patients with bilateral donor sites, the ROM of both sides was averaged.

The existence of perineal herniation was examined by the first author in both the lateral and standing positions. In the standing position, the Valsalva maneuver was performed. Postoperative perineal herniation was defined as the protrusion of intra-abdominal viscera through a defect in the pelvic floor into the perineal region. In the case of perineal herniation, the extent was estimated as the distance to the normal level of the buttocks.

### Data Analysis

The IBM Statistical Package for the Social Sciences (SPSS) version 23 was used for the analyses. Data for the LPF group were compared with the control group using the Mann–Whitney U-test or Chi square test. The effect of the reconstruction on the EORTC QLQ-C30 and CR29 data was determined using linear regression. Treatment (reconstruction or no reconstruction) and all significant differences in study characteristics (tumor classification, excision type, type of [chemo]radiotherapy, and time between surgery and receiving back the survey) (Table 1) were included as fixed variables, and the EORTC scale was included as a random variable. A *p* value < 0.05 was considered statistically significant.

**Table 1:** Study characteristics

	Lotus petal flap group [*n* = 15]	Control group [*n* = 16]	*p*-Value
Age, years [mean (SD)]	60.4 (13.6)	63.7 (9.9)	0.64
Sex, male	12 (80.0)	11 (68.8)	0.47
Tumor type			0.17
Rectal cancer	12 (80.0)	16 (100)	
Anal cancer	2 (13.3)	0 (0)	
Giant condylomata	1 (6.7)	0 (0)	
Tumor classification			0.04
T1	2 (13.3)	0 (0)	
T2	3 (20.0)	8 (50.0)	
T3	6 (40.0)	8 (50.0)	
T4	4 (26.7)	0 (0)	
Excision type			0.001
APE	0 (0)	16 (100)	
ELAPE	4 (26.7)	0 (0)	
ELAPE + excision sacrum	4 (26.7)	0 (0)	
Total exenteration	4 (26.7)	0 (0)	
Total exenteration + excision sacrum	2 (13.3)	0 (0)	
Total colectomy + rectal amputation	1 (6.7)	0 (0)	
Radiotherapy	15 (100)	16 (100)	NA
Type of (chemo)radiotherapy			0.01
Long-course chemoradiation	13 (86.7)	7 (43.8)	
Short-course radiotherapy (5 × 5 Gy)	1 (6.7)	9 (56.3)	
Previous radiotherapy	1 (6.7)	0 (0)	
Time between surgery and survey, months [median (range)]	30.6 (16.4–64.3)	16.1 (13.5–21.2)	<0.001

*APE* abdominoperineal excision, *ELAPE* extralevator abdominoperineal excision, *SD* standard deviation, *NA* statistical analyses could not be performed due to a 100% score in both groups

Data are expressed as *n* (%) unless otherwise specified

Descriptive data on physical and sexual functioning were reported as *n* (%) or median (range). In the FSFI/MFSI questionnaires, the domain scores were considered missing in case more than half of the items of that domain were missing, otherwise missing items were replaced by the mean of the known items.

## Results

### Descriptives

**Fig. 1:**
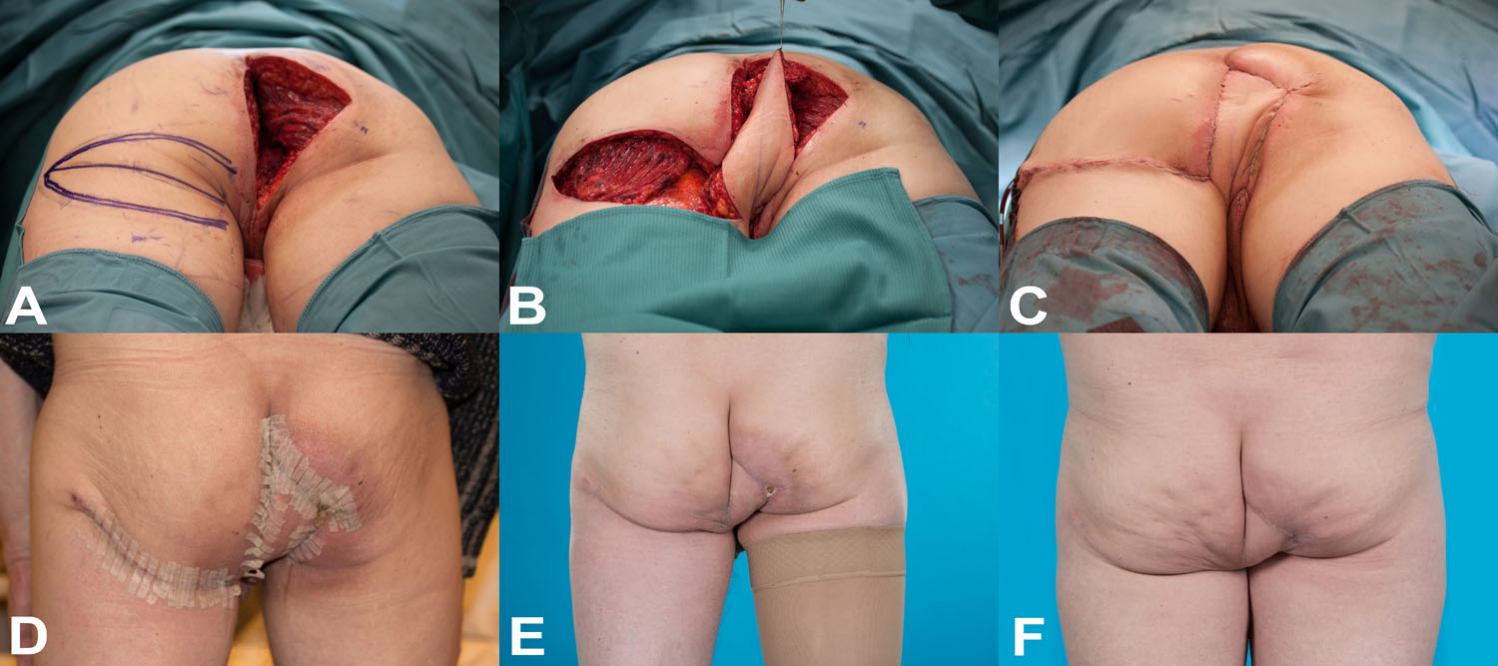
A 55-year-old male patient. (**a**) Defect following ELAPE; (**b**) harvest of lotus petal flap; (**c**) direct post-reconstruction; (**d**) 11 days post-reconstruction; (**e**) 10 weeks post-reconstruction; (**f**) 2 years post-reconstruction. *ELAPE* extralevator abdominoperineal excision

Thirty-five patients underwent reconstruction of a perineal defect with LPF between 2011 and 2015. Eleven patients were deceased at the time of study and one patient was excluded because they were not able to read or write Dutch. In total, 23 patients were asked to participate. Fifteen patients (65.2%) returned the questionnaire and 11 of the 23 patients (47.8%) agreed to participate in the physical examination. Figure 1 shows the pre-, inter- and postoperative result of reconstruction with the LPF in a typical case.

Four patients (26.7%) did not have any postoperative complications; seven patients (46.7%) had a wound dehiscence that healed without further surgical intervention; and four patients (26.7%) had a complication where surgical intervention was required: two patients (13.3%) had partial necrosis of the flap and underwent necrotomy and additional reconstruction with another LPF. The other two patients (13.3%) had an abscess and underwent surgical incision and drainage. No late complications due to radiotherapy were reported.

### Quality of Life

The LPF and control groups did not significantly differ in mean age and sex, but patients in the LPF group had significantly larger tumors and had undergone more extensive treatment (Table 1). One female patient also underwent a posterior vaginal wall reconstruction with the LPF. In the control group, no biological mesh was used, while in the LPF group, both an omentoplasty and biological mesh were used as part of reconstruction of the pelvic floor in 10 patients (66.7%). In three patients (20.0%), an omentoplasty only was used, and, in one patient (6.7%), a biological mesh only was used, leaving one patient (6.7%) with neither an omentoplasty nor a biological mesh. No biological mesh-related complications were reported. The LPF group showed a significantly longer time between surgery and the return of the completed survey (*p* < 0.001).

There were no significant differences between both groups in terms of general QoL scores (EORTC QLQ-C30) and colorectal-specific QoL scores (EORTC QLQ-CR29) [Table 2]. The global health status and some of the functional scales of the EORTC QLQ-C30 (physical functioning, emotional functioning, and cognitive functioning) showed a positive reconstruction effect. Although those differences were not significant in our small study population, it shows a trend towards higher scores of the LPF group, indicating a higher global health status and a higher quality of physical, emotional, and cognitive functioning in the LPF group. The other functional scales of the EORTC QLQ-C30 (role functioning and social functioning) and the functional scales of the EORTC QLQ-CR29 (body image, anxiety, and weight) showed a negative reconstruction effect. This shows a trend towards higher scores of the control group, indicating a higher quality of role and social functioning, higher body image, less anxiety, and less weight problems in the control group; however none of these differences were significant in our small study population.

**Table 2:** EORTC QLQ-C30 and EORTC QLQ-CR29 scores

	Lotus petal flap group [*n* = 15]		Control group [*n* = 16]		Reconstruction effect (*β*)	95% CI	*p*-Value
	Median	Range	Median	Range			
*EORTC QLQ*-*C30*							
Global health status	66.7	33.3–100	79.2	33.3–100	8.0	− 17.0 to 33.0	0.51
Functional scales							
Physical functioning	66.7	26.7–100	80.0	0.0–100	6.7	− 19.3 to 32.7	0.60
Role functioning	50.0	16.7–100	83.3	0.0–100	− 11.5	− 43.8 to 20.7	0.47
Emotional functioning	75.0	25.0–100	95.8	0.0–100	3.7	− 32.0 to 39.4	0.83
Cognitive functioning	83.3	16.7–100	83.3	16.7–100	3.3	− 29.6 to 36.1	0.84
Social functioning	66.7	16.7–100	100	0.0–100	− 6.8	− 44.1 to 30.5	0.71
*EORTC QLQ*-*CR29*							
Functional scales							
Body image	33.3	0.0–100	77.8	0.0–100	− 18.1	− 61.5 to 25.3	0.40
Anxiety	66.7	0.0–100	83.3	0.0–100	− 18.3	− 63.0 to 26.4	0.41
Weight	100	0.0–100	100	0.0–100	− 6.0	− 44.9 to 32.9	0.76

A high score represents a high quality of life or a high level of functioning

*CI* confidence interval, *EORTC QLQ*-*CR29* European Organization for Research and Treatment of Cancer Quality of Life Questionnaire CR29, *EORTC QLQ*-*C30* European Organization for Research and Treatment of Cancer Quality of Life Questionnaire C30

### Sexual Functioning

In the LPF group, 12 of the 15 patients had a partner (80%) and 13 of the 15 patients were sexually active before surgery (86.7%). Five patients (33.3%), one female and four males, reported being sexually active postoperatively; all five were also sexually active preoperatively. Four (26.7%) of these patients, one female and three males, had intercourse after surgery. Of the three patients who completed the FSFI/MSFI, the median total score was 16.8 (15.2–30.1). One patient scored above the cut-off point of 26 for normal sexual functioning.

In the control group, nine patients (56.3%), seven males and two females, were sexually active postoperatively.

### Lotus Petal Flaps-Specific Physical Functioning

Six patients underwent a reconstruction with a unilateral (54.5%) LPF, four of whom reported no limitations in hip function on either side. Two patients reported limitations on both sides during sitting. Five patients underwent a bilateral LPF reconstruction (45.5%), one of whom reported no limitations, two reported limitations on both sides during sitting and activities, and two reported limitations on just one side during activities. All except one of the included patients experienced limitations in the duration of sitting, varying from 1 to 60 min. Five patients were no longer able to ride a bicycle following reconstruction (three for a limited duration, varying from 2 to 60 min), and three patients experienced no limitations during cycling. During physical testing, the ROM of the LPF group had a median maximum flexion of 105° (range 80–130) and a median maximum (hyper)extension of 10° (range 0–25). No signs of perineal herniation were found in any of the 11 patients who underwent reconstruction.

## Discussion

The aim of this study was to evaluate QoL, sexual functioning, and physical functioning following perineal reconstruction using the LPF technique. The main results of our study show that despite the extent of the resection performed in the LPF group, QoL was not significantly different from that of patients with a smaller resection, in whom there was no need for reconstruction using flaps. Earlier studies on perineal reconstruction following resection for rectal cancer compared the use of the vertical rectus abdominis myocutaneous (VRAM) flap with primary closure. Those studies showed no or only small differences between both groups on the EORTC QLQ-C30 and EORTC QLQ-CR29.^23^,^24^ QoL studies in the field of oncology often reveal little changes in QoL, and Sprangers and Schwartz explained this phenomenon as response shift,^25^ which means that a life-threatening disease, such as cancer, may change a patient’s internal standards of self-evaluation. Therefore, the experience with cancer may change their expectations about life and, as such, they may evaluate their QoL according to these new expectations and internal standards, resulting in the report of a relatively good QoL despite the large impact of this major life event.^25^–^27^

The results also indicated that, in retrospect, patients who underwent LPF reported decreased sexual and physical functioning. Moreover, only one patient scored above the cut-off of normal sexual functioning postoperatively. Low sexual functioning could be partially due to the reconstruction, but also the malignant disease itself may have influenced sexual functioning. Low sexual functioning has been reported in earlier studies on preoperative patients diagnosed with colorectal carcinoma, in which the mean and median scores of patients were well below the cut-off of normal sexual functioning.^28^,^29^ No earlier studies on sexual functioning following LPF reconstruction have been reported. An earlier study on postoperative sexual functioning after perineal resection comparing patients without reconstruction with patients who underwent reconstruction using the VRAM technique showed that the non-reconstruction group had slightly better sexual functioning (mean 21.0 vs. 12.9).^30^ It should be noted though that this study, like ours, was performed in a small group (*n* = 29), which limits the reliability of the findings. However, given the consistency in findings, it does stress the importance of discussing sexual functioning during preoperative counseling since the reconstruction seemed to have an impact on sexual functioning. Indeed, in their systematic review on sexual function following perineal reconstruction, McArdle et al.^31^ also emphasized the importance of preoperative counseling to ensure realistic goals and expectations on postoperative sexual functioning.

Regarding LPF-specific physical functioning, many patients reported limitations during sitting, especially those patients after bilateral use of LPF. The median ROM of the hip (105°) was lower compared with the average ROM of healthy people (120°), as published by the American Academy of Orthopaedic Surgeons;^22^ however, this degree of flexion is still considered normal and does not cause any impairment.^32^ In addition, the hip extension was decreased following LPF reconstruction (10°) compared with untreated people (30°).^22^ Hypothetically, the donor site on the posterior side of the hip may cause (temporary) tightness of the skin and limit flexion of the hip. In that case, only a decrease in flexion would have been expected. However, in our study group, limitation during both flexion and extension was seen. The decreased ROM (both flexion and extension) could possibly be due to either the radiotherapy or to pain.

During the outpatient clinic visits, no signs of perineal herniation were found in any of the 11 patients. The incidence of perineal herniation following ELAPE without use of a flap ranges from 1 to 26%. Our outcome is comparable with the systematic review of Balla et al.^12^ on perineal hernia repair after perineal resection, who also reported low recurrence rates of perineal herniation when a flap was used. Use of an omentoplasty or biological mesh also contributes to the prevention of perineal herniation. Reconstruction using the LPF seems to have a limited influence on the ROM and to prevent perineal herniation.

### Limitations

This study had a small study population and a broad range in time between surgery and survey. This is hard to overcome as the application of this reconstruction technique is limited and, in addition, many patients unfortunately have a limited life expectancy following treatment of advanced rectal and anal cancer. Cases in the LPF group had larger defect sizes than those in the control group. This selection bias cannot be avoided. Patients with defects large enough to require reconstruction cannot be closed primarily, and it would be unethical to reconstruct patients when their wound can be closed primarily. However, this is the largest study to date on the subject of QoL and sexual and physical functioning following perineal reconstruction with the LPF. Prospective multicenter studies, with a standardized presurgical assessment on QoL, sexual functioning, and physical functioning, are needed to increase the quality and quantity of data.

## Conclusion

LPF reconstruction after extensive perineal resection did not impair QoL when compared with perineal resection without reconstruction, even though the resection size was much larger in the LPF group. Both sexual and physical functioning become, to a certain extent, impaired following LPF reconstruction in the perineal area, although not hampering overall satisfaction, and no cases of perineal herniation were observed.
